# Innovation Culture Declines Drive Exnovation of Patient Engagement Strategies in Primary Care

**DOI:** 10.1007/s11606-026-10513-4

**Published:** 2026-05-20

**Authors:** Hector P. Rodriguez, Matthew B. Mackwood, Stephen M. Shortell, Elliott S. Fisher, Karen E. Schifferdecker

**Affiliations:** 1 School of Public Health, University of California, Berkeley, Berkeley, CA, USA; 2 The Dartmouth Institute for Health Policy & Clinical Practice, Dartmouth College, Lebanon, NH, USA

**Keywords:** patient engagement, organizational culture, care delivery innovations, shared decision-making, health information technology

## Abstract

**BACKGROUND:**

Patient engagement strategies (PES) are behavioral and relational in nature. They include using patient-reported outcomes (PROs) for treatment monitoring, using shared decision-making (SDM) tools, and training clinicians in motivational interviewing. The long-term maintenance of PES by primary care practices requires substantial frontline effort, adaptation to workflow, and supportive organizational cultures.

**OBJECTIVES:**

To measure the adoption and exnovation of PES by adult primary care practices and to identify organizational factors associated with the count of exnovated PES, defined as the deliberate or passive removal of previously adopted strategies.

**DESIGN:**

Retrospective longitudinal cohort study.

**PARTICIPANTS:**

A national cohort of US adult primary care physician practices (*n* = 714), spanning two waves of the National Survey of Healthcare Organizations and Systems (NSHOS I: 2017–2018 and NSHOS II: 2022–2023).

**MAIN MEASURES:**

The outcome was the number of PES that the practice exnovated, on net. Independent variables included baseline measures and change scores for the practice’s innovation culture and health information technology functionalities.

**KEY RESULTS:**

Practice-level adoption of ten PES increased slightly from a mean of 4.2 (SD = 2.5) to 4.8 (SD = 2.7). Nearly half of practices (46%) exnovated PES on net. A decline in organizational culture of innovation was the strongest predictor of net exnovation (IRR 1.10; 95% CI, 1.06–1.15; *P* < 0.001), followed by an increase in health information technology functionality (IRR 1.11; 95% CI, 1.01–1.22; *P* = 0.03).

**CONCLUSIONS:**

The exnovation of patient engagement strategies is widespread in primary care, driven by declines in organizational cultures that support innovation. The expansion of health information technology may reduce practice capacity to maintain human resources-intensive relational innovations, such as SDM and motivational interviewing, posing a challenge to improving patient-centered primary care.

## INTRODUCTION

Innovation in healthcare delivery is a complex process involving not only the adoption of new practices but also exnovation, the deliberate or passive removal of previously adopted innovations. Exnovation ensures that primary care practices retain sufficient absorptive capacity,^1^ or the ability to recognize, assimilate, transform, and apply new external knowledge to improve patient care. The exnovation process can be driven by the erosion of organizational slack or discretionary resources. These resources, such as flexible staff time and financial reserves, allow an organization to adapt to and maintain complex changes.^2^

Patient-centered care is a cornerstone of high-quality primary care, associated with improved clinical outcomes and patient care experiences.^3^ Patient engagement strategies (PES) are central to supporting active patient participation in care decisions.^4^ PES are delivery system innovations that help transform primary care by supporting patient participation and active engagement in their own care. These strategies, which include the use of patient-reported outcomes (PROs) for treatment monitoring and decision-making,^5-7^ the use of shared decision-making (SDM) tools such as decision aids,^8-11^ and clinician training in motivational interviewing,^12-14^ are behavioral and relational in nature. The Patient Protection and Affordable Care Act’s value-based payment reforms, including the Medicare Shared Savings Program (MSSP), Center for Medicare and Medicaid Innovation (CMMI) projects, and the Patient-Centered Medical Home (PCMH) model, were designed to lower costs and improve patient outcomes,^15^ which incentivized health systems to use PES to achieve these goals.

PES are highly dependent on organizational slack for training, team reinforcement, and workflow adaptation. When organizational slack is crowded out, often by mandated activities such as the expansion of health information technology (HIT), these resource-intensive, patient-centered innovations may be vulnerable to passive or deliberate removal. Indiscriminate or passive exnovation, particularly of high-value patient-centered innovations in primary care, threatens improvements in care quality and patients’ experiences.

Implementing PES requires sustained frontline effort, adaptation to workflow, and a supportive organizational culture. This distinctiveness sets them apart from primarily process-based innovations, such as electronic health record (EHR) registries or chronic care management processes, which are relatively more established and evidence-based practices relative to PES. While strategies like motivational interviewing are long-standing, they are “innovations” in that they have not reached widespread adoption and remain relatively new or only partially implemented in most primary care practices.^16^

Prior research examining chronic care management processes and their exnovation in a cohort of physician practices from 2008 to 2013 found that over one-third (34.1%) of practices were net exnovators.^17^ In that prior context, expansion of HIT functions was protective against exnovation of chronic care management processes, suggesting that HIT capabilities helped institutionalize these evidence-based processes.

Given PES’s heavy reliance on human capital, training, and cultural alignment, and its potential for exnovation, this study analyzes a cohort of US primary care practices to examine the extent to which patient engagement capabilities changed between 2017/2018 and 2022/2023. We hypothesized that the adoption and exnovation dynamics of PES would be more turbulent than those observed for processes that support chronic care management. Additionally, we expected that the COVID-19 pandemic, which occurred during the study period, would place stress on organizational cultures and likely exacerbate the resource constraints and organizational instability that drive exnovation of PES.^18^

Recent research suggests that the cultural orientations of primary care practices toward innovation—such as learning and openness, support for experimentation, and psychological safety—are strongly associated with sustained practice participation in quality improvement (QI)^19^ and robust medical assistant staffing.^20^ Primary care practice cultures of innovation may also affect their ability to retain PES; however, no research has examined the association of organizational culture with exnovation of PES. We posit that declines in primary care practice cultures of innovation will be associated with the exnovation of PES, on net.

## METHODS

### Data Sources and Study Sample

Two waves of the National Survey of Healthcare Organizations and Systems (NSHOS) were integrated and analyzed: Wave 1 (NSHOS I, 2017–2018, response rate (RR) = 47%) and Wave 2 (NSHOS II, 2022–2023, RR = 38%).^19^ Both surveys used a nationally representative sampling frame of US physician practices with three or more primary care physicians based on IQVIA OneKey Data. The survey was completed by a single lead respondent, typically a practice manager or lead physician knowledgeable about the practice’s clinical and administrative operations. For this analysis, a longitudinal cohort of 714 physician practices that responded to both NSHOS I and NSHOS II was created. Survey weights that account for non-response and differential sampling probabilities were used in all analyses. The NSHOS webpage contains full copies of each survey instrument.^21^

### Outcome Measure

Our primary outcome was net PES exnovation, a count variable representing the number of strategies removed, because we were primarily interested in identifying practices experiencing a net loss of patient-centered capacity. It was constructed by (1) calculating the PES change score (Wave 2 count – Wave 1 count), (2) conditionally transforming this score: all practices that experienced net adoption or maintenance (Change Score ≥ 0) were set to a net PES exnovation count of zero because they did not contribute to the net decline. For net exnovators, the count was the absolute count of removed strategies.

The 10 PES items are: four items assessing PRO measures (e.g., depression, disability, and pain for specific conditions), one item assessing SDM training, one item assessing motivational interviewing training, three items assessing decision aid use (for breast cancer, diabetes, and joint knee replacement), and one item assessing use of patient experience/satisfaction data for quality improvement (QI).^16^ Decision aids are tools to help patients gain knowledge and reduce decisional conflict in the face of preference-sensitive conditions.^22,23^ The 10 items were selected because they represent diverse, evidence-based methods of involving patients in their care, ranging from technical (PROs) to relational (motivational interviewing). Appendix, eTable 1 includes item response options and the binary scoring approach used for each item.

### Independent Variables

Our first main independent variable was practice innovation culture. This 7-item measure assessed the shared perceptions of policies, practices, and procedures that a practice expects, supports, and rewards, which are necessary for continuous improvement and successful change implementation. The items assess learning and openness (e.g., team members sharing challenges and failures; publicizing successful innovations), support for experimentation (e.g., time given to generate new ideas; encouraging trying new ideas; acting as a testing ground for new approaches), and psychological safety (e.g., sense of belonging; team members feeling safe raising concerns).^24,25^ Possible answer choices for each question were: Never, Sometimes, Often, or Always. To ease the interpretation of changes in innovation culture over time and to reliably classify the presence of each dimension of innovation culture, we constructed binary indicators for each of the 7 items. To do this, survey responses of “Always” and “Often” were categorized as 1, and other responses were categorized as 0 (range: 0–7 for each year; internal consistency reliability, *α*=0.72).

Our second main independent variable was a 6-item measure of binary HIT functionality questions (range: 0–6; *α* = 0.65). This measure assessed the practice’s ability to connect to a hospital EHR and facilitate patient electronic access to medical records. It also evaluated secure physician-to-patient communication, prescription tracking, and advanced analytical systems such as data mining.^19^ Although the HIT scale is below the 0.70 threshold, it is a valuable measure of technical capacity in this context.

Regression model covariates included practice ownership (including Federally Qualified Health Center [FQHC] status and changes in ownership over time), practice size, geographic region, and baseline Medicaid revenue (≥ 20%), in addition to baseline levels and changes in HIT functionality, accountable care organization (ACO) participation, and two area-level economic factors (baseline and change in county unemployment and poverty rates). The covariates were chosen to account for structural (size, ownership), geographic (region), and socioeconomic (Medicaid revenue, area-level poverty) factors that influence practice resources and stability.

### Statistical Analysis

For descriptive analyses, practices were categorized as (1) net PES exnovators (practices with negative PES change scores), (2) maintainers (practices with no change in PES scores), or (3) net PES adopters (practices with positive PES change scores), and covariates for the three categories were compared using omnibus statistical tests.

We modeled the count of exnovated PES items using a negative binomial regression to account for the count nature and overdispersion of the outcome variable.^26^ In this model, the results are presented as incidence rate ratios (IRRs). An IRR greater than 1.0 indicates an increased rate (or count) of PES exnovation, indicating that the factor is associated with the removal of PES. Conversely, an IRR less than 1.0 indicates a protective factor associated with a lower PES exnovation rate. These models estimated the association of temporal changes in organizational capabilities and the count of net adopted PES.

As supplemental analyses, we examined net adoption of PES by estimating parallel negative binomial regression models. Based on the results of both regression models, we estimated predicted probabilities to illustrate the adjusted associations between practice culture and net PES exnovation and net PES adoption. The Committee for the Protection of Human Subjects, University of California, Berkeley (#2022-01-14941) approved the research study.

## RESULTS

Over the 5 years, primary care practice-level adoption of PES increased slightly from a mean of 4.2 (SD = 2.5) to 4.8 (SD = 2.7). Nearly half (46%) of practices exnovated PES, on net, while 41% were net adopters. Of the 10 patient engagement strategies, practices increased the adoption of seven strategies over time, including all 4 PRO approaches, training in shared decision-making, and the use of decision aids for both diabetes medication selection and breast cancer screening (Appendix eTable 2). Practice-level adoption of three strategies decreased over time, including training in motivational interviewing (51.4% at Time 1 vs. 50.7% at Time 2), decision aids for knee joint replacement (26.7% vs. 17.9%), and the use of patient experience measures for QI (65.1% vs. 46.9%).

Detailed analysis of the individual adoption and exnovation rates for each of the nine strategies is provided in Table 1. The percentages refer to the proportion of practices that reported using the strategy at Wave 1 but not at Wave 2 (exnovation rate). Relational PES, such as the use of patient experience measures for QI (37.1%), decision aids for knee joint replacement (17.0%), and motivational Interviewing training for clinicians and staff (15.4%), had the highest exnovation rates (Table 1).

The mean NSHOS II PES count for net exnovators was 3.0 (standard deviation (SD): 1.8), a decline from their NSHOS I baseline score of 4.8 (SD: 2.4). Conversely, net adopters increased from 3.4 (SD: 2.4) to 6.8 (SD: 2.2) (Table 1).

Net exnovators were most likely to be FQHCs (58.8%) and solo practices (52.1%) (Table 2). In contrast, net adopters were most likely to be independent practices (53.1%) and practices owned by healthcare systems (16.5%). Regarding practice capabilities, net exnovators were characterized by a high proportion of moderate or high Medicaid revenue (70.2%) at baseline. While net exnovators had a higher mean innovation culture at baseline (5.4, SD: 1.5), they subsequently experienced a mean decline in innovation culture (−2.9, SD: 2.3), whereas adopters started at a lower baseline level (3.5, SD: 2.1) and experienced an improvement (0.6, SD: 2.4) (*P* < 0.001).

Item-level analyses of HIT functions reveal a complex dynamic regarding the practice’s HIT capabilities (Table 3). On average, exnovators of PES had a slight increase in HIT functions (from 3.5 to 3.7). In contrast, the mean HIT functions for net adopters of PES actually declined (from 3.3 to 2.3). For net exnovators, the highest adoption rate (57.5%) was for the function “EHR allows patients to comment/input information to their medical records,” indicating an investment in basic, scalable technological tools for patient engagement.

Multivariable regression analysis confirmed the primary associations with net PES exnovation found in descriptive analyses. A decline in the practice’s change in innovation culture was the strongest predictor of net exnovation of PES (IRR = 0.84, 95% CI: 0.78–0.91, *P* < 0.001) (Table 4). A practice’s baseline level of HIT functionality was associated with a modest protective effect against exnovation (IRR 0.93, 95% CI 0.86–0.99, *P* < 0.05). However, expansion in HIT functionality was associated with a significantly increased rate of PES exnovation (IRR, 1.11; 95% CI, 1.01–1.22; *P* < 0.05). No other covariates were significantly associated with net exnovation of PES in adjusted analyses.

Appendix eTable 3 presents negative binomial regression analyses examining the adoption of PES. These results indicate that many more covariates were significantly associated with net PES adoption, including health system ownership, an improved practice innovation culture, baseline HIT functionality, increases in HIT functionality, and increases in ACO contract participation.

**Figure 1:**
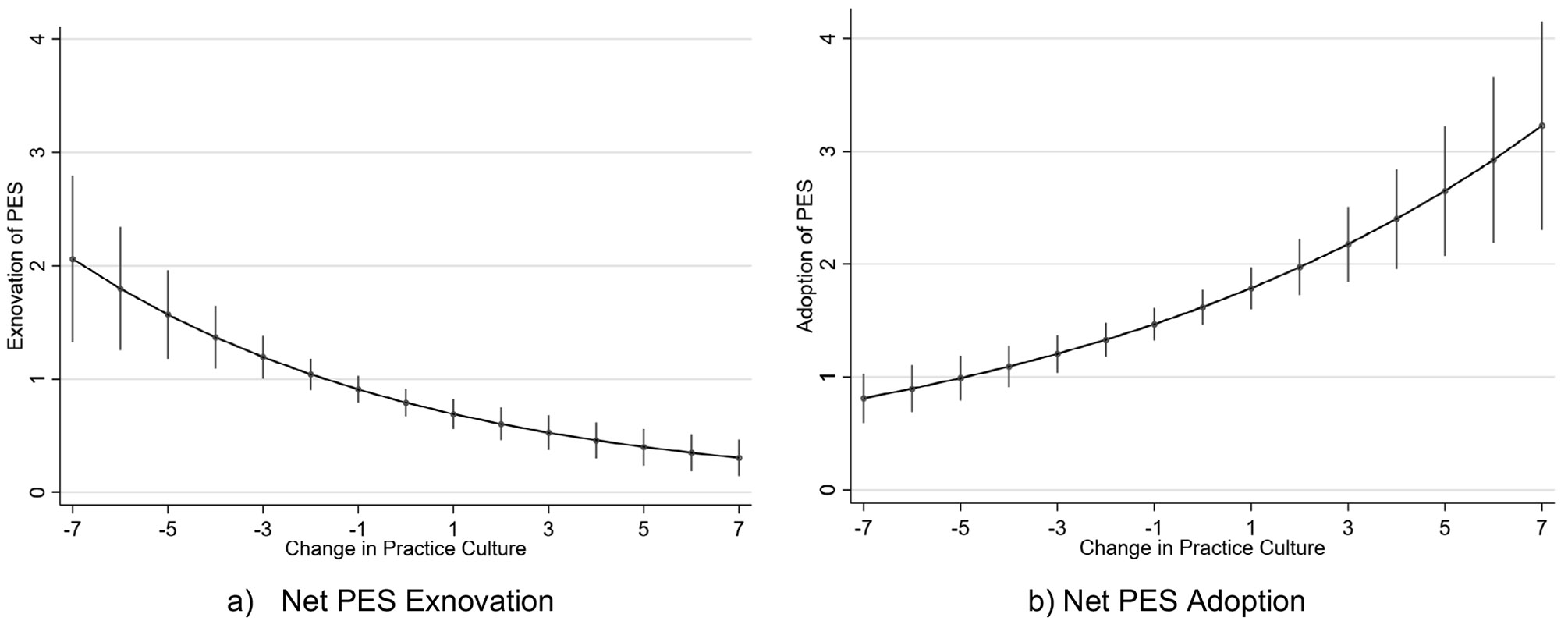
Predicted net PES exnovation and net PES adoption, by change in practice innovation culture. (a) Net PES exnovation. (b) Net PES adoption

The relationships between practice culture and net PES adoption, as well as practice culture and net PES exnovation, are detailed in Fig. 1. As the figure illustrates, net PES exnovators experienced a decline in innovation culture while net PES adopters experienced an improvement.

## DISCUSSION

Although practice-level adoption of 10 patient engagement strategies increased slightly (from 4.2 to 4.8) over 5 years, 41% of adult primary care practices were net adopters. In contrast, almost half (46%) were net exnovators, underscoring the challenge of maintaining relational innovations in primary care. The 46% net exnovation rate for PES is substantially higher than the previously documented 34% rate for chronic care management processes.^17^ The net exnovators reduced their use of ten PES from an average of 4.8 at baseline to 3.0 at follow-up, indicating a significant loss of patient-centered capabilities. Exnovation of breast cancer decision aids, in particular, may be a response to evolving clinical guidelines (e.g., changes by the U.S. Preventive Services Task Force) rather than a loss of capacity.^27^ The overall patterns, however, suggest that the primary challenge for the dissemination of PES is maintaining innovation rather than organizational adoption.

Expanded HIT functionality was associated with greater PES exnovation, contrasting with its protective effect on care management processes.^17^ This “HIT paradox” suggests that technological expansion may hinder rather than aid the maintenance of relational innovations. The difference in the findings may relate to the characteristics of the innovations being examined. Chronic care management processes (e.g., patient registries, clinical reminders) are primarily technical, automated, and embedded within EHR functionality. PES vary in their resource demands, and the strategies most vulnerable to exnovation are human-intensive, relational strategies that require high organizational slack, such as motivational Interviewing training and using patient experience data for QI. These strategies are relational and behavioral processes that are fundamentally human-intensive and not easily automated. HIT implementation or upgrades can divert critical organizational slack from other areas. This often displaces non-technical resources, such as team training. Consequently, practices may lose the capacity to maintain relational PES and clinician feedback.^28^

In primary care practices, non-billable, human-intensive activities, such as motivational interviewing and shared decision-making, may be especially vulnerable to displacement. For example, a practice that adopts advanced predictive analytics may shift staff focus to data collection and data quality, leaving less time for the “high-touch” requirements of SDM or motivational interviewing. Research evidence suggests that this displacement is often driven by “technostress,” in which the cognitive load of navigating sophisticated digital interfaces reduces clinicians’ capacity for the patient-centered communication approach required by PES.^29,30^ The automation of patient communication, for example, the use of standardized portal messages, may also contribute to the de-prioritization of individualized human outreach. Technology can support efficiency, but it can also substitute for relational care if not implemented within a team-based care context with sufficient staff capacity to engage patients.

HIT and PES are not inherently opposed; in fact, EHR-integrated decision aids can facilitate SDM.^31^ Our findings, however, suggest that expanding HIT functions may displace human effort needed to maintain PES. PRO implementation may initially be technical; sustained use for QI (e.g., using patient experience measures for QI, which had a high exnovation rate) depends on non-technical organizational slack. Relational innovations require significant non-reimbursed time for training and workflow adaptation.

Our observation that a decline in innovative practice cultures is the strongest predictor of a decrease in PES supports the proposition that, without cultural backing, the discretionary nature of these strategies makes them vulnerable to elimination. The high exnovation risk we found for PES may be especially pronounced in primary care practices, given their limited capacity to absorb change.^32^ The COVID-19 pandemic likely exacerbated the resource constraints and cultural declines leading to exnovation.

Systematic PES removal may impact clinician burnout. De-implementing strategies after significant training investment creates operational burdens and dissatisfaction.^33^ Insufficient integration can lead to frustration and moral injury, further contributing to professional burnout.^34,35^ When primary care practices face high burnout, clinicians may experience “cognitive narrowing” where they instinctively prioritize high-stakes clinical tasks and mandatory documentation over elective, high-touch engagement activities like SDM or motivational interviewing.^36,37^ Exnovation of PES may serve as an early warning sign of systemic burnout within a practice. Maintaining successful patient-centered capabilities is therefore not just a concern about quality of care but may also help retain the primary care clinician workforce.

FQHCs did not have an increased risk of net PES exnovation in adjusted analyses, likely due to controlling for size, culture change, and Medicaid revenue. However, FQHCs experienced greater innovation culture declines than non-FQHCs, leaving them structurally vulnerable to resource shifts.

The fact that highly vetted innovations like SDM are being exnovated points to the continuing problem with dominant payment models in health care. SDM adoption has been slow due to overworked physicians, insufficient training, and a lack of consistent methods to measure and reward the time and effort required.^38,39^ CMS attempted to address these barriers through targeted alternative payment models. The CMS SDM Model, for instance, was designed to test how to integrate a structured four-step SDM process into routine clinical practice, directly addressing the time and resource gaps.^40^ The CMS model pays participating ACOs $50 for each SDM engagement, covering the discussion, decision, and documentation.^41^ It also funds non-reimbursable administrative support, such as identifying beneficiaries and distributing decision aids. These payments provide the organizational slack needed for successful adoption and maintenance. This type of payment reform, which integrates financial incentives for the non-clinical effort required for high-touch, relational patient engagement, may be needed to protect PES from being crowded out and subsequently exnovated by physician practices.

Team-based care may provide organizational slack to help maintain PES in primary care practices. For instance, when motivational interviewing or the administration of PRO measures is integrated into care team members’ workflows, the cognitive load on physicians is reduced, thereby preventing PES exnovation. High turnover among frontline staff can disrupt the organizational memory required to maintain PES. By formalizing care team member responsibilities for patient engagement and promoting cultures of innovation, primary care practices can ensure that these patient-centered strategies remain a standard of care.

## Limitations

Our study has limitations, including reliance on self-reported data, which is subject to reporting bias; the inability to measure the quality or fidelity of PES implementation; and the inability to determine whether exnovation was passive or deliberate. The extended study period (2017/2018 to 2022/2023) encompasses the entire COVID-19 pandemic, so the study design does not permit attributing exnovation of PES to specific pandemic-related events. Furthermore, our focus on a longitudinal cohort of practices that responded to both survey waves may introduce sampling bias, as these practices may be more stable than non-respondents. Regression to the mean could also partially explain the exnovation patterns. The association with specific organizational changes, however, suggests exnovation reflects actual organizational dynamics rather than measurement imprecision.

## Conclusion

The exnovation of PES by primary care practices is common, significantly impacting the maintenance of relational innovations in primary care. This disinvestment is influenced by declines in organizational culture, which may be compounded by resource competition and constraints due to HIT expansion. To maintain patient-centered care, mitigate equity gaps in primary care delivery, and reduce professional burnout, interventions and policies should focus not only on adopting new practices but also on building and protecting the organizational culture and slack necessary to make human-intensive, relational innovations resilient against resource competition. Value-based payment models that finance the human effort and non-reimbursable time required to engage patients are a promising path forward. To prevent widening disparities, payment reforms should also account for the maintenance costs of primary care practices required to engage socioeconomically vulnerable patients.

## Supplementary Material

Supplementary Material

**Supplementary Information** The online version contains supplementary material available at https://doi.org/10.1007/s11606-026-10513-4.

## Figures and Tables

**Table 1:** Adoption and Exnovation of Patient Engagement Strategies Between 2017/2018 and 2022/2023, by Net Practice Changes Between 2017/2018 and 2022/2023

	All practices		Net PES exnovators		Net PES adopters		PES maintainers	
*N* (% of practices)	714		329 (46% of practices)		292 (41% of practices)		93 (13%)	
	Mean (SD)		Mean (SD)		Mean (SD)		Mean (SD)	
2017/2018 PES composite score^1^	4.2 (2.5)		4.8 (2.4)		3.4 (2.4)		4.9 (2.5)	
2022/2023 PES composite score^1^	4.8 (2.7)		3.0 (1.8)		6.8 (2.2)		4.9 (2.5)	
	% Adopting	% Exnovating	% Adopting	% Exnovating	% Adopting	% Exnovating	% Adopting	% Exnovating
1. Depression PROs	12.8	2.7	3.8	5.6	25.1	0.2	6.0	0.3
2. Disability PROs for older adult patients	44.2	7.5	55.4	12.5	32.5	2.6	41.0	5.2
3. Pain PROs for diabetic patients	46.0	12.2	54.0	22.8	48.6	2.9	8.8	4.0
4. Pain PROs for knee joint replacement	48.8	11.5	54.9	21.4	55.4	3.1	6.2	2.6
5. Motivational interviewing training for clinicians/staff	14.7	15.4	4.1	12.5	26.3	7.9	15.8	48.9
6. Training in shared decision-making	26.7	8.5	13.3	13.1	36.9	3.3	41.9	8.9
7. Decision aids for breast cancer screening	15.0	11.1	3.3	22.5	32.5	1.2	1.3	1.7
8. Decision aids for diabetes	30.1	7.0	8.7	14.3	61.1	0.4	7.9	1.4
9. Decision aids for knee joint replacement	8.7	17.0	0.6	21.5	20.4	2.0	0.5	48.1
10. Use of patient experience measures for quality improvement	19.1	37.1	5.3	70.2	37.9	7.7	8.7	12.5

1 PES composite score is the sum of 10 patient engagement strategies (potential range: 0–10)

*PES*, patient engagement strategies; *PROs*,patient-reported outcome measures

See Appendix, eTable 1 for detailed information about each question

**Table 2:** Practice Characteristics and Area-Level Factors, by Practice Changes (2017/18 vs. 2022/23) in Patient Engagement Strategies

	All practices (*n* = 714)	Net PES exnovators (*n* = 329, 46%)	Net PES adopters (*n* = 292, 41%)	Net PES maintainers (*n* = 93, 13%)	*P*-value
Ownership at baseline					^‡^
Independent (%)	37.1	22.7	53.1	37.8	
Hospital-owned (%)	5.9	6.2	6.3	3.2	
Medical group-owned (%)	7.8	4.8	10.2	10.7	
Health care system-owned (%)	16.0	7.5	16.5	44.4	
Federally qualified health center (%)	33.3	58.8	13.9	3.9	
Ownership change (%)	11.3	7.6	14.8	13.7	^†^
Practice size at baseline					^‡^
Single physician (%)	25.7	52.1	4.0	0.3	
2–9 physicians (%)	54.3	37.6	63.3	85.0	
10–19 physicians (%)	16.1	7.6	28.2	7.9	
20+ physicians (%)	3.9	2.7	4.5	6.8	
Change in practice size (mean, SD)	0.5 (3.2)	0.6 (2.6)	0.5 (4.0)	0.3 (2.3)	
Practice capabilities					
Innovation culture at baseline [0–7] (mean, SD)	4.5 (2.0)	5.4 (1.5)	3.5 (2.1)	4.1 (2.1)	^‡^
Change in innovation Culture (mean, SD)	−1.1 (2.8)	−2.9 (2.3)	0.6 (2.4)	−0.6 (1.7)	^‡^
Moderate or high Medicaid revenue (≥ 20%) at baseline (%)	44.7	70.2	25.9	13.4	^‡^
Change in % of moderate/high Medicaid revenue	−0.8	−1.9	−1.4	5.4	^‡^
Practice involved in ACO contracts at baseline (mean, SD)	1.4 (1.9)	0.7 (1.5)	1.5 (2.0)	3.2 (1.8)	^‡^
Change in ACO contracts (mean, SD)	0.5 (2.1)	1.5 (2.0)	−0.3 (1.7)	−0.7 (1.8)	^‡^
Area-level factors					
US census region					^‡^
Midwest (%)	11.3	7.8	16.6	7.0	
Northeast (%)	14.4	5.7	13.4	48.7	
South (%)	31.2	26.0	37.7	29.4	
West (%)	43.0	60.5	32.3	14.9	
Rural-urban commuting area (RUCA) codes					^†^
Metropolitan (%)	89.0	89.1	86.1	98.0	
Micropolitan (%)	7.7	7.1	10.7	0.8	
Small town (%)	2.2	3.0	1.9	0.7	
Rural (%)	1.0	0.8	1.4	0.6	
% 18–64 without health insurance at baseline	11.4 (4.5)	11.2 (4.4)	12.2 (4.1)	10.1 (5.4)	^‡^
Change in % uninsured	−2.1 (2.2)	−2.1 (2.0)	−2.3 (2.4)	−1.6 (1.9)	*
Unemployment rate (%), 16+ at baseline	4.2 (1.0)	4.1 (1.0)	4.3 (1.0)	4.3 (1.0)	^†^
Change in % unemployment	1.0 (1.1)	1.4 (1.0)	0.8 (1.2)	0.9 (0.9)	^‡^
% in poverty at baseline	29.5 (7.7)	29.8 (7.0)	30.1 (7.3)	24.2 (9.0)	^‡^
Change in % poverty	9.1 (4.2)	9.2 (4.0)	9.6 (4.3)	6.9 (4.1)	^‡^

Net PES exnovators are practices with negative PES change scores. PES maintainers are primary care practices with no change in PES scores. Net PES adopters are practicing with positive PES change scores

^‡^, ^†^, and * indicate significance *P* < 0.001, *P* < 0.01, *P* < 0.05, respectively

*SD*, standard deviation; *ACO*, accountable care organization; *PES*, patient engagement strategies

**Table 3:** Adoption and Exnovation of Individual Health Information Technology Functions, by Practice Changes (2017/2018 vs. 2022/2023) in Patient Engagement Strategies

	All practices		Net PES exnovators		Net PES adopters		PES maintainers	
*N* (% of practices)	714		329 (46% of practices)		292 (41% of practices)		93 (13% of practices)	
	Mean (SD)		Mean (SD)		Mean (SD)		Mean (SD)	
2017/2018 HIT composite score	3.4 (1.5)		3.5 (1.3)		3.3 (1.7)		3.4 (1.7)	
2022/2023 HIT composite score	3.2 (1.8)		3.7 (1		2.3 (1.9)		4.2 (1.9)	
HIT function	% Adopting	% Exnovating	% Adopting	% Exnovating	% Adopting	% Exnovating	% Adopting	% Exnovating
1. EHR connects directly to the EHR at the main hospital	7.7	10.0	5.5	5.2	8.8	4.8	11.6	43.3
2. EHR allows patients electronic access to medical records	15.9	4.1	1.1	4.6	33.8	4.8	12.1	0.3
3. EHR allows patients to comment/input information to their medical records	37.2	15.6	57.5	17.6	16.0	5.3	32.2	41.4
4. EHR allows physician-to-patient communications via secure messaging	10.1	4.8	1.6	4.6	20.3	4.8	8.2	5.5
5. EHR allows physicians to know whether patient filled prescriptions	26.0	37.3	8.3	58.8	47.9	12.2	20.0	40.0
6. EHR allows for advanced analytic systems (predicting utilization, data mining, etc.)	17.6	14.1	10.1	10.5	24.4	9.2	22.4	42.1

Net PES exnovators are practices with negative PES change scores. PES maintainers are primary care practices with no change in PES scores. Net PES adopters are practicing with positive PES change scores. *HIT*,health information technology; *EHR*, electronic health record

**Table 4:** Negative Binomial Regression Model of the Net Exnovation of Patient Engagement Strategies (2017/2018 to 2022/2023)

	Net PES exnovation	
	Incidence rate ratio (IRR)	95% CI
Ownership at baseline		
Independent (reference)	-	-
Medical group-owned	1.29	0.87–1.90
Hospital-owned	1.13	0.75–1.71
Health care system-owned	1.13	0.79–1.60
Federally qualified health center	0.68	0.37–1.24
Ownership change		
Stable ownership (reference)	-	-
Ownership change—more integrated	1.19	0.85–1.64
Practice size at baseline		
Single physician	1.2	0.51–2.81
2–9 physicians	1.58	1.00–2.50
10–19 physicians	1.39	0.85–2.29
20+ physicians (reference)	-	-
Region		
West	1.1	0.75–1.61
Midwest	0.79	0.56–1.11
Northeast	0.61*	0.40–0.91
South (reference)	-	-
Practice capabilities		
Revenue from Medicaid (≥ 20%) in baseline	1.02	0.75–1.39
Practice culture at baseline	1.04	0.96–1.14
Change in practice Culture	0.87^‡^	0.82–0.93
HIT functionality at baseline	0.99	0.88–1.11
Change in HIT functionality	1.11*	1.01–1.22
ACO contracts at baseline	0.96	0.86–1.06
Change in ACO contracts	0.94	0.85–1.04
Area-level factors		
Unemployment (%) in baseline	0.99	0.88–1.13
Change in % unemployment	0.99	0.88–1.11
Poverty (%) in baseline	0.98	0.95–1.01
Change in % poverty	0.97	0.91–1.04
Inflation portion		
Solo physician	0.11	0.00–1,210.48
2–9 physicians	1.94	0.72–5.27
10–19 physicians	0.84	0.26–2.66
20+ physicians (reference)	-	-
Constant	2.28	0.95–5.52
Observations	714	

^‡^ and * indicate significance *P* < 0.001 and *P* < 0.05, respectively

*PES*, patient engagement strategies; *ACO*, accountable care organization; *HIT*, health information technology

An IRR greater than 1.0 indicates an association with increased net exnovation (i.e., a larger negative change score, or removal of strategies). An IRR less than 1.0 indicates a protective effect against net PES exnovation

Regression results for Net PES adoption are reported in the Appendix (eTable 4)

## Data Availability

Full copies of the survey instrument are available on the NSHOS webpage: https://sites.dartmouth.edu/coe/nshos/. Limited research data can be requested by contacting the NSHOS PI listed on the website.
