# Supplementary material for: Innovation Culture Declines Drive Exnovation of Patient Engagement Strategies in Primary Care
Source: J Gen Intern Med. Author manuscript; Available in PMC 2026 Jun 18. (PMC13277227; doi:10.1007/s11606-026-10513-4)
Supplement: Supplementary Material [file NIHMS2181495-supplement-Supplementary_Material.docx]

**eTable 1. Adoption and Exnovation of Patient Engagement Strategies Between 2017/18 and 2022/23, Net Adopters and Maintainers of Patient Engagement Strategies**

| **Question stem** | **Focus of Question** | **Response Options** | **Binary Scoring for the PES Count score for each wave** |
| --- | --- | --- | --- |
| 1. Does your practice collect patient-reported measures of | Depression | Yes; No | "Yes"=1, "No"=0 |
| 1. Does your practice collect patient-reported measures of | Physical function or disability for older adult patients | Yes; No | "Yes"=1, "No"=0 |
| 1. Does your practice collect patient-reported measures of | Pain for diabetic patients | Yes; No | "Yes"=1, "No"=0 |
| 1. Does your practice collect patient-reported measures of | Pain for knee joint replacement patients | Yes; No | "Yes"=1, "No"=0 |
| 1. Does your practice have clinicians/staff who are formally trained in motivational interviewing? |  | Yes, clinicians only.  Yes, staff only.  Yes, both clinicians and staff.  No | Any of the "Yes" options=1, "No"=0 |
| 1. Considering the clinicians in your practice, how many | Are formally trained in shared decision-making | None; Some; Most; All | "All" or "Most"=1, "Some" or "None"=0 |
| 1. Considering the clinicians in your practice, how many | Routinely use decision aids for breast cancer screening | None; Some; Most; All | "All" or "Most"=1, "Some" or "None"=0 |
| 1. Considering the clinicians in your practice, how many | Routinely use decision aids for initial treatment of diabetes | None; Some; Most; All | "All" or "Most"=1, "Some" or "None"=0 |
| 1. Considering the clinicians in your practice, how many | Routinely use decision aids for knee joint replacement | None; Some; Most; All | "All" or "Most"=1, "Some" or "None"=0 |
| 1. How does your practice use information about individual clinician performance for | Patient experiences (e.g., patient satisfaction or CAHPS scores): Use for feedback | Check if applicable | Checked= 1, Not checked=0 |

**eTable 2. Practice-Level Availability of Patient Engagement Strategies, 2017/2018 vs. 2022/2023**

| **Patient Engagement Strategy (% of Practices Available)** | **Overall (n=714)** | | **Net PES Exnovators (n=329, 46%)** | | **Net PES Adopters (n=292, 41%)** | | **Net PES Maintainers (n=93, 13%)** | |
| --- | --- | --- | --- | --- | --- | --- | --- | --- |
|  | Time 1 | Time 2 | Time 1 | Time 2 | Time 1 | Time 2 | Time 1 | Time 2 |
| 1. Depression PROs | 85.7 | 94.6 | 95.1 | 90.8 | 72.8 | 97.7 | 92.7 | 98.3 |
| 1. Disability PROs for older adult patients | 40.8 | 52.1 | 39.3 | 27.3 | 39.4 | 69.3 | 50.0 | 85.7 |
| 1. Pain PROs for diabetic patients | 40.6 | 48.9 | 40.2 | 17.8 | 39.4 | 84.2 | 45.3 | 48.0 |
| 1. Pain PROs for knee joint replacement | 39.5 | 49.8 | 41.8 | 20.4 | 34.5 | 83.5 | 46.9 | 48.2 |
| 1. Motivational Interviewing Training for Clinicians / Staff | 51.4 | 50.7 | 72.3 | 63.9 | 24.8 | 43.2 | 60.8 | 27.7 |
| 1. Training in Shared Decision-Making | 22.5 | 40.6 | 23.3 | 23.5 | 20.9 | 54.6 | 24.3 | 57.3 |
| 1. Decision aids for breast cancer screening | 28.0 | 31.8 | 30.7 | 11.5 | 23.4 | 54.6 | 32.7 | 32.3 |
| 1. Decision aids for diabetes (0,1) | 21.8 | 44.9 | 23.8 | 18.2 | 18.0 | 78.7 | 26.6 | 33.1 |
| 1. Decision aids for knee joint replacement (0,1) | 26.2 | 17.9 | 22.9 | 2.0 | 18.0 | 36.5 | 63.5 | 15.8 |
| 1. Use of patient experience measures for quality improvement (0,1) | 65.1 | 46.9 | 87.8 | 22.7 | 44.3 | 74.2 | 50.7 | 46.9 |

Note: Time 1= 2017/2018; Time 2= 2022/2023

**eTable 3. Negative Binomial Regression Model of the Net Adoption of Patient Engagement Strategies (2017/2018 to 2022/2023)**

|  | **Net PES Adoption** | |
| --- | --- | --- |
|  | IRR | 95% CI |
| **Ownership at baseline** |  |  |
| Independent (reference) | - | - |
| Medical Group-owned | 1.1 | 0.85 - 1.43 |
| Hospital-owned | 1.23 | 0.88 - 1.72 |
| Health care system-owned | **1.43**** | **1.14 - 1.81** |
| FQHC | 1.19 | 0.85 - 1.66 |
| **Ownership change** |  |  |
| Stable ownership (reference) | - | - |
| Ownership change - more integrated | 0.97 | 0.74 - 1.28 |
| **Practice size at baseline** |  |  |
| Single physician | 1.06 | 0.47 - 2.39 |
| 2-9 physicians | 1.18 | 0.88 - 1.60 |
| 10-19 physicians | 1.26 | 0.87 - 1.81 |
| 20+ physicians (reference) | - | - |
| **Region** |  |  |
| West | 0.81 | 0.62 - 1.06 |
| Midwest | 0.82 | 0.65 - 1.02 |
| Northeast | 0.92 | 0.71 - 1.18 |
| South (reference) | - | - |
| **Practice capabilities** |  |  |
| Revenue from Medicaid (>= 20%) in baseline | 0.82 | 0.67 - 1.00 |
| Practice Culture at baseline | 1.02 | 0.97 - 1.08 |
| Change in Practice Culture | **1.10***** | **1.06 - 1.15** |
| HIT functionality at baseline | **0.93*** | **0.86 - 0.99** |
| Change in HIT functionality | **0.89***** | **0.84 - 0.95** |
| ACO contracts at baseline | 1.04 | 0.97 - 1.12 |
| Change in ACO contracts | **1.09*** | **1.02 - 1.17** |
| **Area-level factors** |  |  |
| Unemployment (%) in baseline | 1.04 | 0.95 - 1.15 |
| Change in % unemployment | 1 | 0.92 - 1.08 |
| Poverty (%) in baseline | 1.02 | 0.99 - 1.04 |
| Change in % poverty | 0.97 | 0.93 - 1.01 |
| **Inflation portion** |  |  |
| Solo physician | 3.22 | 0.72 - 14.31 |
| 2-9 physicians | 0.95 | 0.47 - 1.93 |
| 10-19 physicians | 2.14 | 0.95 - 4.84 |
| 20+ physicians (reference) | - | - |
| Constant | 1.62 | 0.88 - 2.97 |
| Observations | 714 | |

Note: ***, **, and * indicate significance p<0.001, p<0.01, p<0.05, respectively;

An IRR greater than 1.0 indicates a positive association with net PES adoption (a larger positive change score). An IRR less than 1.0 indicates a negative association with net PES adoption.

ACO= accountable care organization; HIT=health information technology;

**eTable 4. Practice Innovation Culture and HIT Changes, by FQHC status**

|  | **All Practices** | **FQHCs** | **Non-FQHCs** | **p-value** |
| --- | --- | --- | --- | --- |
| Change in Practice Culture | -1.1 (2.8) | -2.6 (2.7) | -0.4 (2.6) | 0.000*** |
| Change in HIT | -0.2 (1.9) | -0.1 (1.1) | -0.3 (2.2) | 0.304 |
